# Effective Practices for Enrolling Underrepresented Groups in Early Alzheimer’s Disease Trials: Insights from the Aducanumab Program

**DOI:** 10.1002/alz.095729

**Published:** 2025-01-09

**Authors:** Shardae Showell, Jennifer Murphy, Cai Gillis, Nancy N Maserejian, Sangeetha Mayuram, Jenna Wilson, Aimee Farrell, Katie King, Savine DaCosta, Kate Wilson, Kerry Lovelace, Andrew Vigario, Katherine Dawson, Gersham Dent

**Affiliations:** ^1^ Biogen, Cambridge, MA USA

## Abstract

**Background:**

Alzheimer’s disease is a progressive neurodegenerative disorder characterized by cognitive decline, memory loss, and impairment of daily functioning. It disproportionately affects certain racial and ethnic groups, with African Americans and Hispanics being 2 and 1.5 times more likely to develop Alzheimer’s disease compared to white non‐Hispanic Americans, respectively. Despite higher disease prevalence in these groups, enrolling these patients in Alzheimer’s disease clinical trials has been challenging, and they have historically been underrepresented. For the aducanumab program, we developed strategies to boost enrollment of African American and Hispanic participants. Here, we share insights from employing these strategies to further advance the enrollment of underrepresented groups in Alzheimer’s disease clinical trials.

**Method:**

To boost enrollment of African American and Hispanic participants, we developed a cross‐functional work stream, incorporating members from clinical development, epidemiology, clinical operations, patient engagement, clinical trial diversity, and clinical country site management to collaborate on diverse recruitment and enrollment strategies. We executed and measured the impact of these strategies through the screening, enrollment, and retention of participants from these groups.

**Result:**

The creation of intentional recruitment practices and site‐led strategies increased screening and randomization of underrepresented populations. Successful strategies included brain awareness education programs and faith‐based initiatives to build a trusting relationship with communities, mobile research unit initiatives to combat access barriers, and tailored site‐led programs that engaged populations in specific geographic/demographic locations. In parallel, targeted site communications and study‐wide webinars provided rationale for the goals and strategies of the trials, explaining the critical scientific need to include biological data from diverse populations.

**Conclusion:**

Employing a wide range of operational interventions, incentivizing strategies, outreach initiatives, and site educational events enhanced the recruitment and enrollment of underrepresented populations in an aducanumab clinical study. Clinical trials that are representative of the overall Alzheimer’s disease population may aid in producing results that better reflect real world outcomes.

